# Productivity-adjusted life years lost due to type 2 diabetes in Germany in 2020 and 2040

**DOI:** 10.1007/s00125-021-05409-3

**Published:** 2021-03-04

**Authors:** Thaddäus Tönnies, Annika Hoyer, Ralph Brinks

**Affiliations:** 1 Institute for Biometrics and Epidemiology, German Diabetes Center (DDZ), Leibniz Center for Diabetes Research at Heinrich Heine University, Düsseldorf, Germany; 2 Department of Statistics, Ludwig Maximilians University, Munich, Germany; 3 Chair for Medical Biometry and Epidemiology, Witten/Herdecke University, Faculty of Health/School of Medicine, Witten, Germany

**Keywords:** Burden of disease, Prevalence, Productivity, Projection, Type 2 diabetes, Years of life lost

## Abstract

**Aims/hypothesis:**

Type 2 diabetes can lead to reduced productivity during working age. We aimed to estimate productive life years lost associated with type 2 diabetes on the individual and population level in Germany in 2020 and 2040, while accounting for future trends in mortality.

**Methods:**

Based on a mathematical projection model, we estimated age- and sex-specific productivity losses associated with type 2 diabetes during working age (20–69 years) in Germany in 2020 and 2040. Productivity losses in terms of excess mortality (years of life lost, YLL) and reductions in labour force participation, presenteeism and absenteeism (years of productivity lost, YPL) were summed to calculate productivity-adjusted life years (PALY) lost. Input data for the projection were based on meta-analyses, representative population-based studies and population projections to account for future trends in mortality.

**Results:**

Compared with a person without type 2 diabetes, mean PALY lost per person with type 2 diabetes in 2020 was 2.6 years (95% CI 2.3, 3.0). Of these 2.6 years, 0.4 (95% CI 0.3, 0.4) years were lost due to YLL and 2.3 (95% CI 1.9, 2.6) years were lost due to YPL. Age- and sex-specific results show that younger age groups and women are expected to lose more productive life years than older age groups and men. Population-wide estimates suggest that 4.60 (95% CI 4.58, 4.63) million people with prevalent type 2 diabetes in 2020 are expected to lose 12.06 (95% CI 10.42, 13.76) million PALY (1.62 million years due to YLL and 10.44 million years due to YPL). In 2040, individual-level PALY lost are projected to slightly decrease due to reductions in YLL. In contrast, population-wide PALY lost are projected to increase to 15.39 (95% CI 13.19, 17.64) million due to an increase in the number of people with type 2 diabetes to 5.45 (95% CI 5.41, 5.50) million.

**Conclusions/interpretation:**

On the population level, a substantial increase in productivity burden associated with type 2 diabetes was projected for Germany between 2020 and 2040. Efforts to reduce the incidence rate of type 2 diabetes and diabetes-related complications may attenuate this increase.

**Graphical abstract:**

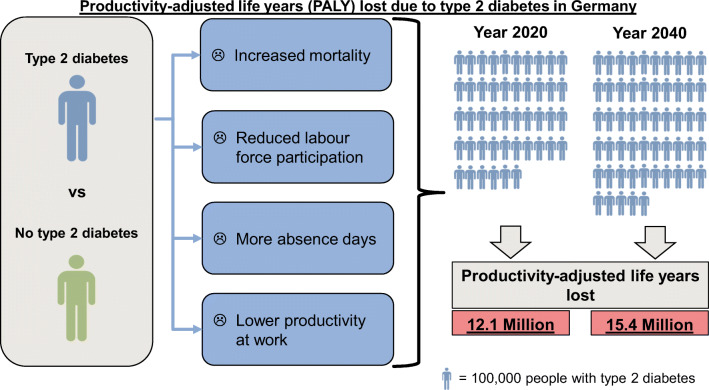

**Supplementary Information:**

The online version of this article (10.1007/s00125-021-05409-3) contains peer-reviewed but unedited supplementary material..

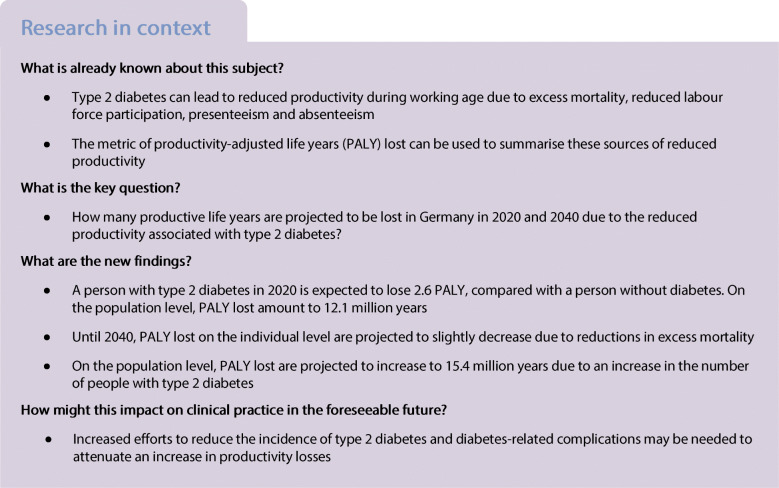



## Introduction

People with diabetes suffer not only from macro- and microvascular complications as well as reduced health-related quality of life, but also from reduced productivity during working age, which imposes an economic burden on individuals and populations [1–3]. Besides physical impairments, declines in cognitive functioning associated with type 2 diabetes can potentially reduce productivity [4, 5]. Hence, compared with people without diabetes, reduced productivity with type 2 diabetes is caused by an increased number of sick leave days (absenteeism), reduced productivity while at work (presenteeism) and reduced labour force participation (LFP) [1, 6]. Although absenteeism, presenteeism and/or LFP were monetised as indirect costs in several previous cost-of-illness studies [3, 7, 8], few studies estimated the individual and population-wide consequences for productivity in terms of productive life years [9, 10]. For instance, using the metric of productivity-adjusted life years (PALY), Magliano et al. [10] estimated that productive life years were reduced by approximately 11% among the population with diabetes in Australia in 2011.

In light of substantial future increases in the number of people with diabetes globally, projecting future productivity losses seems warranted to inform decision makers and people with diabetes. In addition, most European countries are facing substantial population ageing, leading to a decreasing proportion of people of working age [11]. Maintaining welfare systems for an increasing proportion of people of retirement age in future decades will therefore be imposed on a decreasing proportion of the population [11]. Within Europe, Germany is one of the countries that will be most affected by this demographic change [11]. A projected increase in the number of people with type 2 diabetes by 54% to 77% in Germany until 2040 [12] has the potential to amplify consequences for economic and welfare systems. These considerations highlight the need to quantify future productivity losses associated with diseases like diabetes, since these are potentially avoidable.

Hence, in this study we aimed to estimate PALY lost associated with type 2 diabetes on the individual and population level in Germany in 2020. Furthermore, we aimed to project PALY lost for the year 2040.

## Methods

Based on a projection model, we estimated individual and population-wide productivity losses associated with type 2 diabetes in Germany in 2020 and 2040. We chose the year 2040 because the proportion of the population in retirement age is projected to increase in Germany particularly during this period [13]. Another reason was that a previous projection study conducted in the context of the German Diabetes Surveillance chose the same period [12]. Furthermore, choosing 2040 has the advantage that accounting for future birth rates in the model was not necessary, because people born after 2020 will be under the age of 20 years in 2040.

On the individual level, we compared one person of a given age (in the range 20–69 years, assuming that retirement age in Germany will increase from 67 to 69 years over next decades) with type 2 diabetes with a person of the same age without type 2 diabetes regarding their remaining life course until age 69 years. The productivity loss of the person with type 2 diabetes consisted of: (1) losses due to excess mortality associated with type 2 diabetes (years of life lost, YLL); and (2) losses due to morbidity (years of productivity lost, YPL). For (2), three components were considered: absenteeism, presenteeism and reduced LFP. PALY lost were defined as the sum of YLL and YPL. The projection model ran until the year 2089, in order to allow estimation of YLL, YPL and PALY lost until age 69 years for a person who was 20 years old in 2040.

For population-wide productivity losses in 2020 and 2040, individual productivity losses of all people with type 2 diabetes in 2020 and 2040 in the age range 20–69 years were summed. To achieve this, we projected the prevalence and number of people with type 2 diabetes for the years 2020 and 2040 based on prevalence data from 2015. All analyses performed were age and sex specific.

### Projection of type 2 diabetes prevalence

We used an illness–death model to project the prevalence and number of people with type 2 diabetes in 2020 and 2040 (details in the electronic supplementary material [ESM] Methods section ‘Projection of type 2 diabetes prevalence’ and ESM Fig. 1) [12]. This model divides the population into the three states: ‘healthy’ (with respect to type 2 diabetes), ‘diabetes’ and ‘dead’. The transition rates between these states are the incidence rate *i* (transition from ‘healthy’ to ‘diabetes’) and the mortality rates for people without and with type 2 diabetes (transitions from ‘healthy’ to ‘dead’ and from ‘diabetes’ to ‘dead’, respectively). The proportion of the population in the ‘diabetes’ state is the prevalence of type 2 diabetes. Due to data availability, we used the mortality rate *m* of the general population and the mortality rate ratio (*MRR*) of people with vs without type 2 diabetes as input values for the partial differential equation that describes the illness–death model, instead of the absolute mortality rates of people with and without type 2 diabetes. This yields the following equation [14]:

$$ \partial p=\left(1-p\right)\times \left[i-\frac{p\times \left( MRR-1\right)\times m}{p\times \left( MRR-1\right)+1}\right], $$

where *p* is the prevalence and *∂p* is the temporal change in prevalence. All model parameters were allowed to vary by age. Furthermore, since our aim was to project future prevalence, all parameters depended on calendar time to incorporate assumed future trends in the incidence rate, the general mortality rate and the *MRR*. To calculate the number of people with type 2 diabetes in 1 year age groups, the population projections of the Federal Statistical Office for 2020 and 2040 were multiplied with the age-specific prevalence from our projection model.

To obtain the future age-specific prevalence, the partial differential equation was solved by integration using nationally representative input data. We used the age-specific prevalence of type 2 diabetes in the year 2015 observed among all people with the German statutory health insurance (*N* ≈ 70 million) as the initial prevalence (ESM Fig. 2) [15]. The age-specific incidence rate (ESM Fig. 3) and *MRR* (ESM Fig. 4) were based on similar data from the years 2012 and 2014 [16, 17]. Unfortunately, the incidence rate and *MRR* were not differentiated by type 1 and type 2 diabetes. Nevertheless, these data serve as a reasonable approximation for the incidence rate of type 2 diabetes, since 90% of cases are estimated to be of this type and the incidence rate of type 1 diabetes is highest in people younger than 20 years [18, 19], further diminishing the proportion of incident type 1 cases in our considered age range. Likewise, the *MRR* is mainly driven by deaths among people with type 2 diabetes. The mortality rate of the general population between 2015 and 2089 (ESM Fig. 5) was based on population projections by the Federal Statistical Office [20]. Current evidence suggests that the mortality rate of people with type 2 diabetes decreases faster than the mortality rate of people without type 2 diabetes, resulting in a decreasing *MRR* [21, 22]. Hence, between 2015 and 2089, we assumed annual decreases of the *MRR* by 0.60% and 0.72% among women and men, respectively, based on evidence from Denmark [21]. Trends in the age-specific incidence rate are heterogeneous [23] and not available for Germany [24]. Hence, for the main analysis we assumed that the age-specific incidence rate remained as observed by Schmidt et al. [16]. In sensitivity analyses, we assumed annual increases and decreases of the age-specific incidence rate by 0.5%, as was done by Tönnies et al. [12].

### Calculation of productivity losses on the individual and population level

The mortality component of productivity loss (YLL) was defined as the difference in remaining life expectancy between a person without and a person with type 2 diabetes, up to age 69. Specifically, life expectancy between a given age *a* and age 69 for a person with type 2 diabetes was subtracted from the life expectancy of a same-aged person without type 2 diabetes. Formally, this measure of YLL is defined by

$$ YLL\left(t,a\right)={\int}_0^{70-a}{S}_{D-}\left(t+u,a+u\right)-{S}_{D+}\left(t+u,a+u\right)\mathrm{d}u, $$

where *S*_*D*−_ and *S*_*D*+_ are the survival functions of people without and with type 2 diabetes, *t* is the calendar time, *a* is age and d*u* is the differential of the integration variable *u*. This measure of YLL can be interpreted as the potential gain in productive life years for a person with type 2 diabetes, if the mortality rate were equal to a person of the same age without type 2 diabetes.

The morbidity component of productivity loss (YPL) for a person with type 2 diabetes consisted of three components. First, we assumed that LFP among people with type 2 diabetes was between 7% and 25% (depending on age and sex) lower than among people without type 2 diabetes, based on evidence from a meta-analysis (ESM Fig. 6) [3] (details on the definition of LFP are available in the ESM Methods section ‘Calculation of productivity losses on the individual and population level’). Second, we assumed that people with type 2 diabetes participating in the labour force have between 1.3 and 1.4 (depending on age and sex) more absence days from work than people without type 2 diabetes (ESM Fig. 6) [3]. Assuming that there are 255 working days per year and 31 paid leave days in Germany [25], this amounts to 0.6% of additional annual working days lost, compared with people without type 2 diabetes. Third, we assumed that productivity while at work (presenteeism) is reduced by 0.4% to 0.5% (depending on age and sex) among people with compared with people without type 2 diabetes (ESM Fig. 6) [3]. We summarised the percentage of work days lost (absenteeism) and the reduced productivity at work (presenteeism) into a productivity loss weight (PLW), by adding up both measures.

YPL for a person with type 2 diabetes were quantified by weighting each year until age 69 years with regard to differences in LFP (ΔLFP), absenteeism and presenteeism while accounting for survival probability. Similar to Hird et al. [9] and Magliano et al. [10], YPL were calculated by:

$$ YPL\left(t,a\right)={\int}_0^{70-a}{S}_{D+}\left(t+u,a+u\right)\times \Delta LFP\left(t+u,a+u\right)+{S}_{D+}\left(t+u,a+u\right)\times \left[1-\Delta LFP\left(t+u,a+u\right)\Big]\times PLW\left(t+u,a+u\right)\right]\mathrm{d}u $$

The first part of the equation calculates the proportion of a year that is lost due to reduced LFP (ΔLFP) while accounting for survival probability (*S*_*D+*_) up to that year. The second part of the equation calculates the proportion of a year that is lost due to absenteeism and presenteeism (PLW), while accounting for the survival probability (*S*_*D+*_) and for the probability to participate in the labour force (1 – ΔLFP), assuming that absenteeism and presenteeism are irrelevant for people outside of the labour force. This measure of YPL can be interpreted as the potential gain in productive life years for a person with type 2 diabetes, if LFP, absenteeism and presenteeism were equal to a person of the same age without type 2 diabetes. Finally, PALY lost were the sum of YLL and YPL.

For the population level, individual age-specific YLL, YPL and PALY were summed over all people with type 2 diabetes in 2020 and 2040. This measure of population-level productivity loss associated with type 2 diabetes can be interpreted as the potential gain in productivity in the population with prevalent type 2 diabetes until age 69 years, if the mortality and productivity of people with and without type 2 diabetes were equal.

### Probabilistic sensitivity analyses

To account for uncertainty in the input data, the procedures described above were repeated 1000 times to get confidence bounds for the point estimates. In each repetition, age-specific input values were randomly drawn from standard normal distributions scaled to the standard errors of the input values. From the resulting 1000 estimates for YLL, YPL and PALY lost, we report the median (2.5th and 97.5th percentile) as the point estimate (95% CI).

## Results

### Productive life years lost on the individual level

**Fig. 1:**
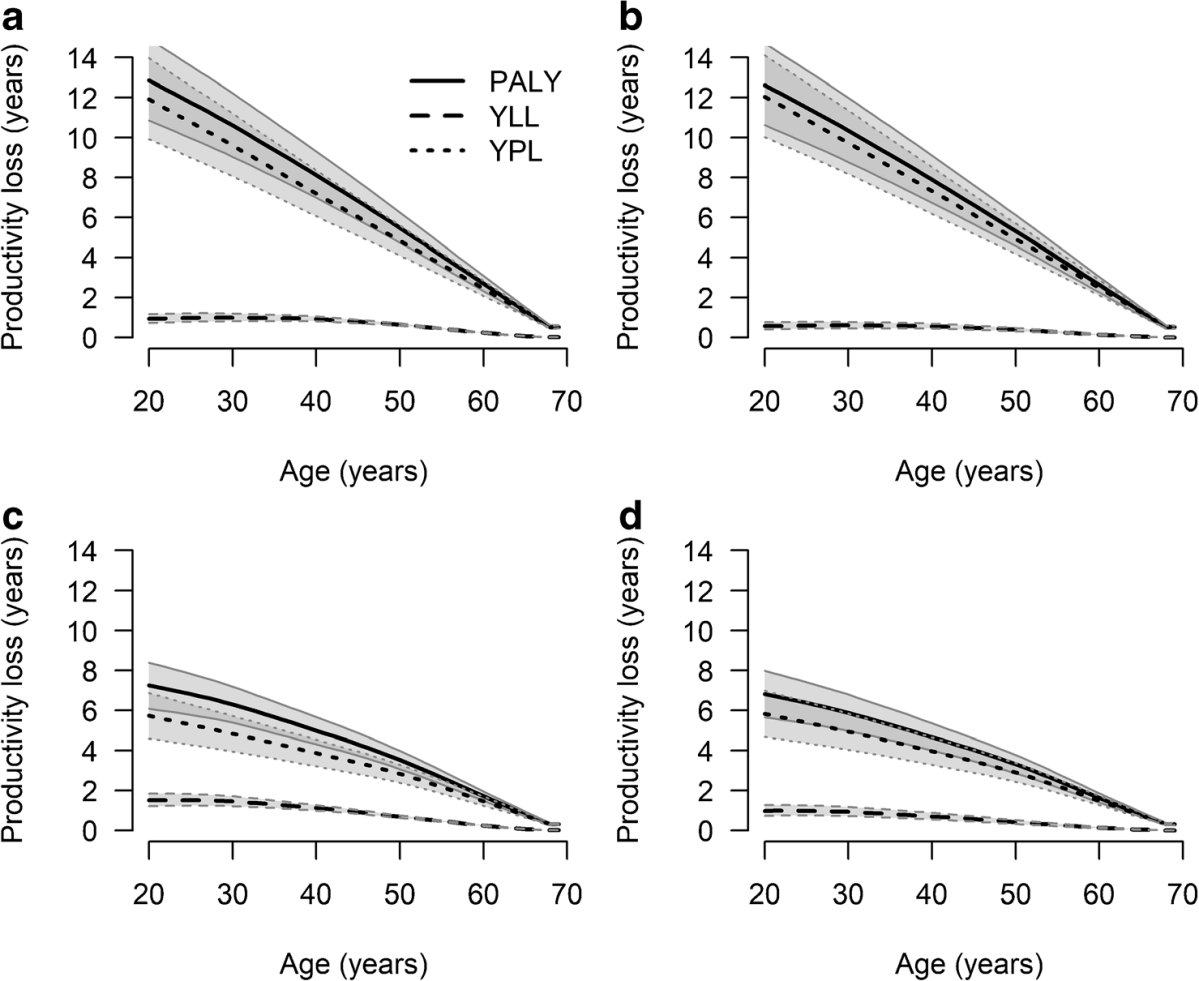
Age-specific PALY lost, YLL and YPL among women (**a**, **b**) and men (**c**, **d**) with type 2 diabetes in 2020 (**a**, **c**) and 2040 (**b**, **d**) compared with people of the same age without type 2 diabetes. Shaded areas with borders corresponding to the key in the figure represent 95% CIs. Overlapping CIs are indicated with darker shading

The highest expected loss in productive life years in the year 2020 was observed among young women. Compared with women without type 2 diabetes, women with type 2 diabetes at age 20 years are expected to lose 12.8 (95% CI 10.9, 14.9) PALY (Fig. 1). Of these 12.8 PALY lost, 11.9 (95% CI 9.9, 14.0) years were due to presenteeism, absenteeism and reduced LFP (YPL) and 0.9 (95% CI 0.7, 1.2) years were due to excess mortality (YLL) (Fig. 1). Since PALY lost, YPL and YLL are measures of absolute productive life time lost, all three measures decreased with increasing age, as the time until age 69 years decreases (Fig. 1). Among men, PALY lost and YPL in the year 2020 were lower compared with women, whereas YLL were higher than among women. The trends over age were similar, resulting in the smallest estimated loss of productive life years for men at age 69 (PALY = 0.3 [95% CI 0.3, 0.4]) (Fig. 1). In the year 2040, PALY lost are projected to be slightly lower among women and men due to reductions in YLL, whereas YPL remained unchanged (Fig. 1).

The age-specific results in Fig. 1 show that in the cohort of people with type 2 diabetes in Germany in 2020, mean PALY lost per person was 2.6 years (95% CI 2.3, 3.0) (Table 1). This number is projected to slightly increase to 2.8 PALY (95% CI 2.4, 3.2) in 2040.

**Table 1:** Mean productive life years lost due to excess mortality (YLL), reduced LFP, absenteeism and presenteeism (YPL) associated with type 2 diabetes

Group	2020	2040
Women		
No. of people with T2D (in 1000s)	1914 (1904, 1923)	2352 (2333, 2372)
YLL	0.3 (0.3, 0.4)	0.2 (0.2, 0.3)
YPL	3.0 (2.6, 3.5)	3.5 (2.9, 4.0)
PALY lost	3.4 (2.9, 3.9)	3.7 (3.2, 4.3)
Men		
No. of people with T2D (in 1000s)	2691 (2679, 2703)	3100 (3075, 3126)
YLL	0.4 (0.3, 0.4)	0.3 (0.2, 0.3)
YPL	1.7 (1.4, 2.0)	1.9 (1.6, 2.2)
PALY lost	2.1 (1.8, 2.4)	2.1 (1.8, 2.5)
Overall		
No. of people with T2D (in 1000s)	4604 (4583, 4626)	5452 (5407, 5499)
YLL	0.4 (0.3, 0.4)	0.2 (0.2, 0.3)
YPL	2.3 (1.9, 2.6)	2.6 (2.2, 3.0)
PALY lost	2.6 (2.3, 3.0)	2.8 (2.4, 3.2)

Numbers are mean (95% CI) productive life years lost per person with type 2 diabetes (T2D) compared with a person of the same sex and age without type 2 diabetes

PALY lost are the sum of YLL and YPL

T2D, type 2 diabetes

**Fig. 2:**
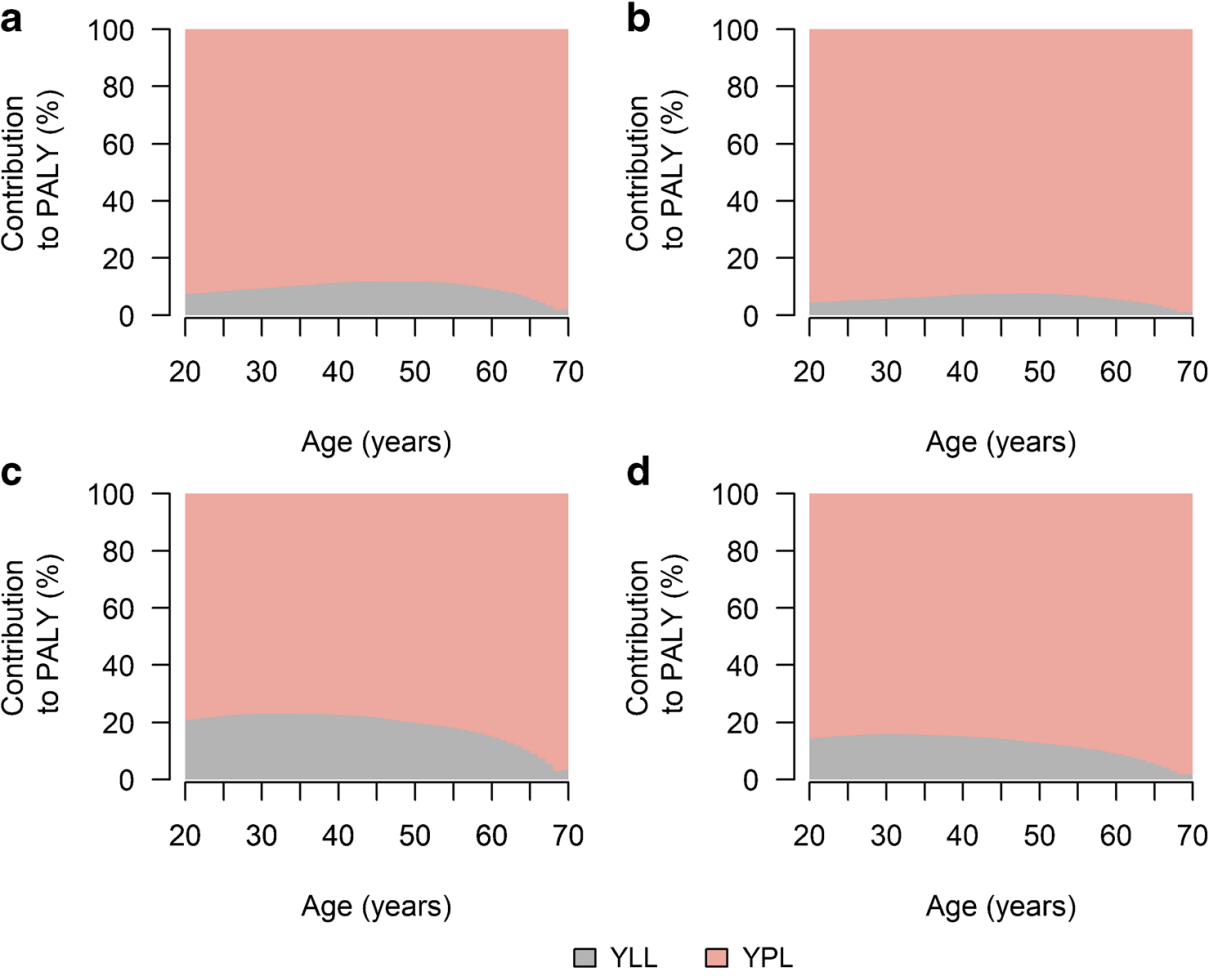
Age-specific relative contributions of YLL and YPL to PALY lost among women (**a**, **b**) and men (**c**, **d**) in 2020 (**a**, **c**) and 2040 (**b**, **d**)

From Fig. 1 it is obvious that YPL contributes more lost productive life years to PALY lost than YLL. More specifically, among women in 2020, YLL contributed approximately between 5% and 15% to PALY lost, depending on age (Fig. 2). Among men, the relative contribution of YLL was higher, exceeding 20% in some age groups. In 2040, YLL contributions were considerably lower among women and men.

**Fig. 3:**
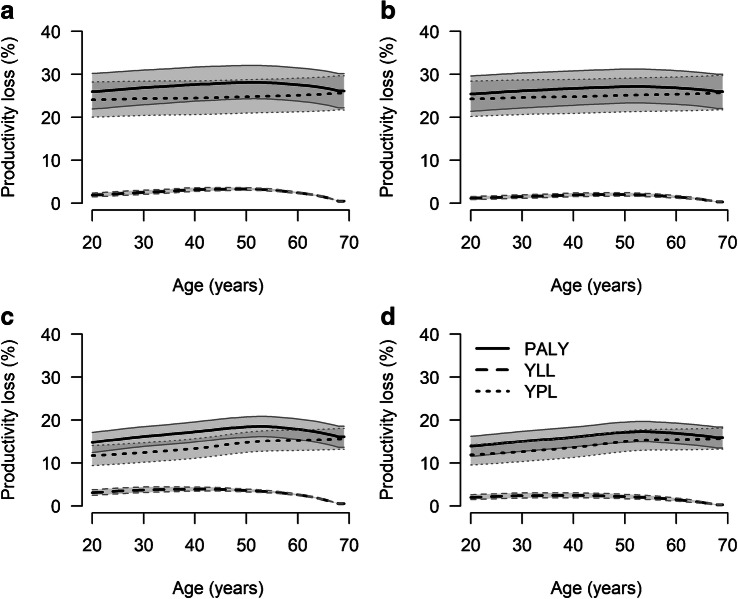
Age-specific percentage of productive life years lost associated with type 2 diabetes compared with no type 2 diabetes among women (**a**, **b**) and men (**c**, **d**) in 2020 (**a**, **c**) and 2040 (**b**, **d**). Shaded areas with borders corresponding to the key in the figure represent 95% CIs. Overlapping CIs are indicated with darker shading

To express the loss in productivity in relative terms, we calculated the percentage of productive life years lost, compared with same-aged people without type 2 diabetes. Over the whole age range, women with type 2 diabetes in 2020 were estimated to experience approximately 25% fewer productive life years than same-aged women without type 2 diabetes (Fig. 3). Among men, percentage of productive life years lost ranged between 15% and 20%, depending on age. In 2040, results were similar to 2020, suggesting that people with type 2 diabetes are not projected to approach the amount of productive life years of people without type 2 diabetes, despite decreasing excess mortality.

Assuming annual increases and decreases of the incidence rate by 0.5% in sensitivity analyses had no influence on the individual-level results.

### Productive life years lost on the population level

To estimate PALY lost, YLL and YPL on the population level, the age-specific results on the individual level for 1 year age groups were multiplied with the projected number of people with type 2 diabetes in 1 year age groups. The results are summarised in Table 2. In 2020, 4.60 (95% CI 4.58, 4.63) million people with prevalent type 2 diabetes in the age range 20–69 years are expected to lose 12.06 (95% CI 10.42, 13.76) million PALY until age 69 (1.62 million years due to YLL and 10.44 million years due to YPL). In 2040, these numbers were projected to increase to 5.45 (95% CI 5.41, 5.50) million people with type 2 diabetes losing 15.39 (95% CI 13.19, 17.64) million PALY (1.33 million years due to YLL and 14.06 million years due to YPL).

**Table 2:** Population-level productive life years lost due to excess mortality (YLL), reduced LFP, absenteeism and presenteeism (YPL) among people with type 2 diabetes of working age in 2020 and 2040

Age group	2020			2040		
	YLL	YPL	PALY	YLL	YPL	PALY
Women						
20–29	0.05 (0.04, 0.06)	0.50 (0.42, 0.58)	0.54 (0.46, 0.63)	0.05 (0.04, 0.06)	0.90 (0.76, 1.06)	0.95 (0.80, 1.11)
30–39	0.09 (0.08, 0.11)	0.79 (0.67, 0.92)	0.88 (0.76, 1.01)	0.10 (0.07, 0.12)	1.40 (1.18, 1.63)	1.49 (1.27, 1.72)
40–49	0.15 (0.13, 0.17)	1.15 (0.97, 1.33)	1.30 (1.12, 1.49)	0.16 (0.13, 0.19)	1.98 (1.68, 2.30)	2.13 (1.82, 2.45)
50–59	0.25 (0.23, 0.27)	2.06 (1.74, 2.38)	2.30 (1.99, 2.64)	0.18 (0.14, 0.21)	2.43 (2.07, 2.82)	2.61 (2.24, 2.99)
60–69	0.09 (0.09, 0.10)	1.33 (1.13, 1.54)	1.42 (1.22, 1.63)	0.06 (0.05, 0.07)	1.47 (1.25, 1.70)	1.53 (1.31, 1.76)
**20–69**	**0.63 (0.56, 0.70)**	**5.82 (4.93, 6.74)**	**6.45 (5.55, 7.40)**	**0.54 (0.43, 0.67)**	**8.19 (6.93, 9.51)**	**8.73 (7.44, 10.04)**
Men						
20–29	0.06 (0.05, 0.07)	0.21 (0.17, 0.25)	0.27 (0.23, 0.31)	0.07 (0.05, 0.09)	0.40 (0.32, 0.47)	0.47 (0.39, 0.54)
30–39	0.13 (0.12, 0.16)	0.45 (0.37, 0.53)	0.59 (0.51, 0.67)	0.13 (0.10, 0.17)	0.72 (0.59, 0.84)	0.85 (0.73, 0.98)
40–49	0.24 (0.22, 0.27)	0.90 (0.76, 1.05)	1.15 (1.00, 1.30)	0.24 (0.19, 0.30)	1.47 (1.24, 1.71)	1.71 (1.47, 1.95)
50–59	0.42 (0.38, 0.45)	1.94 (1.65, 2.25)	2.35 (2.05, 2.66)	0.26 (0.20, 0.33)	2.10 (1.78, 2.43)	2.37 (2.04, 2.69)
60–69	0.14 (0.13, 0.15)	1.12 (0.95, 1.30)	1.26 (1.09, 1.43)	0.08 (0.06, 0.10)	1.19 (1.01, 1.37)	1.27 (1.09, 1.45)
**20–69**	**0.99 (0.90, 1.10)**	**4.63 (3.90, 5.38)**	**5.61 (4.88, 6.37)**	**0.79 (0.61, 0.99)**	**5.88 (4.95, 6.82)**	**6.66 (5.72, 7.61)**
Overall						
20–29	0.10 (0.09, 0.13)	0.70 (0.59, 0.83)	0.81 (0.69, 0.93)	0.12 (0.09, 0.16)	1.30 (1.08, 1.53)	1.42 (1.20, 1.65)
30–39	0.23 (0.19, 0.26)	1.24 (1.04, 1.46)	1.47 (1.26, 1.68)	0.23 (0.18, 0.29)	2.12 (1.77, 2.47)	2.35 (2.00, 2.70)
40–49	0.39 (0.35, 0.44)	2.05 (1.73, 2.39)	2.44 (2.12, 2.78)	0.40 (0.31, 0.49)	3.45 (2.91, 4.01)	3.85 (3.30, 4.40)
50–59	0.66 (0.61, 0.72)	4.00 (3.39, 4.63)	4.66 (4.04, 5.29)	0.44 (0.35, 0.54)	4.54 (3.85, 5.25)	4.98 (4.28, 5.68)
60–69	0.23 (0.22, 0.25)	2.45 (2.08, 2.84)	2.69 (2.30, 3.07)	0.14 (0.11, 0.17)	2.66 (2.26, 3.08)	2.80 (2.39, 3.21)
**20–69**	**1.62 (1.46, 1.80)**	**10.44 (8.82, 12.15)**	**12.06 (10.42, 13.76)**	**1.33 (1.04, 1.65)**	**14.06 (11.88, 16.35)**	**15.39 (13.19, 17.64)**

Numbers are in million years (95% CIs)

PALY lost are the sum of YLL and YPL

Assuming annual increases and decreases of the incidence rate by 0.5% in sensitivity analyses affected the population-level results due to a projected higher and lower number of people with type 2 diabetes (ESM Table 1).

## Discussion

Using an illness–death model, we estimated the productivity burden associated with type 2 diabetes in Germany in 2020 and 2040 from an individual and population perspective. Input data for the illness–death model were based on best available evidence from previous studies. Mean PALY lost per person with type 2 diabetes in Germany in 2020 was 2.6 years until age 69 years due to excess mortality, reduced LFP, presenteeism and absenteeism. Depending on age and sex, PALY lost per person with type 2 diabetes ranged between 0.3 years (men at age 69) and 12.8 years (women at age 20). For people with type 2 diabetes in the year 2040, these numbers were projected to remain similar, with the exception of decreased YLL. In contrast, on the population level, substantial increases in PALY lost were projected due to an increased number of people with type 2 diabetes. In 2020, 4.6 million people with type 2 diabetes aged 20–69 years are expected to lose 12.1 million PALY until age 69, while in 2040, 5.5 million people with type 2 diabetes are projected to lose 15.4 million PALY until age 69. YLL contributed relatively little to overall PALY lost compared with YPL.

Using a life table approach, a comparable study estimated that the cohort of people with diabetes in Australia in 2011 lost 0.79 million productive life years, equating to 1.4 PALY lost per person or an 11% reduction in productive life years compared with no type 2 diabetes [10]. This study ignored period trends in mortality and used a productivity index, which considered presenteeism and absenteeism, but not differences in LFP. Since mortality will most likely decrease in the future, ignoring trends in mortality may overestimate YLL and PALY lost. On the other hand, ignoring LFP may underestimate PALY lost, particularly since evidence suggests that LFP contributes most to reductions in productivity (ESM Fig. 6). Accordingly, our estimates of mean PALY lost per person for Germany were higher (2.6 years vs 1.3 years) compared with Australia. A Chinese study also used a life table approach, but accounted for future trends in mortality and used the same meta-analysis as inputs for reduced LFP, presenteeism and absenteeism that we considered [9]. The results suggest that 75.8 million PALY were lost in the cohort with diabetes in China in 2017, equating to 1.3 PALY lost per person or a 15.1% reduction compared with no diabetes. These results were also lower than ours, probably because Hird et al [9] discounted future life years and calculated PALY only until ages 49 (women) and 59 (men). Furthermore, differences in mean PALY lost could be due to different age structures of the populations with diabetes in Australia, China and Germany. We are not aware of studies that projected future PALY lost associated with diabetes. However, in line with our projected increase in population-level PALY lost, global indirect costs on the population level due to absenteeism, presenteeism, reduced LFP and excess mortality have been projected to increase from 0.46 trillion US dollars in 2015 to 0.73 trillion US dollars in 2030 [26].

Our results have implications for people with type 2 diabetes and for decision makers in healthcare and health policy. For people with type 2 diabetes, 15% to 25% fewer productive life years compared with no diabetes will probably lead to reduced income, imposing a substantial economic burden. The lower income during working age is likely to be maintained in the long term due to reduced federal retirement benefits and private savings. In the context of social epidemiology, our findings can be interpreted in light of the health selection hypothesis, which states that poorer health leads to lower social positions (e.g. measured with income) [27, 28]. However, social position is also associated with type 2 diabetes incidence, which can be interpreted as evidence for the somewhat competing social causation hypothesis [27, 28]. Since there is evidence for both hypotheses, reduced productivity after onset of type 2 diabetes may reinforce health inequalities on the population level, by negatively impacting income and occupation (health selection). Moreover, this may lead to worse diabetes-related outcomes as previous studies suggest that lower social position is associated with an increased risk of diabetes-related complications (social causation) [29]. Vice versa, the presence of complications may lead to an even larger reduction in productivity as some studies show (health selection) [30–32], suggesting a feedback loop between social causation and health selection among people with type 2 diabetes.

With regard to future economic burden, our results suggest that PALY lost on the individual level will not change substantially until 2040, despite assumed future decreases in excess mortality. This is mainly due to the fact that excess mortality contributes relatively little to PALY lost, compared with morbidity (reduced LFP, presenteeism and absenteeism). Hence, for individuals with type 2 diabetes, a focus on preventive efforts that reduce impacts on LFP, presenteeism and absenteeism is needed to attenuate the economic burden of the disease. For instance, preventing diabetes-related complications might be one way to achieve this, since it has been shown that measures of productivity are associated with the presence of complications [30–32]. Similarly, optimising glucose control may attenuate productivity losses caused by reduced cognitive functioning, since it has been shown that hyperglycaemia is associated with reductions in attention and memory [33].

In contrast to the individual perspective, the population level lays a stronger focus on preventing incident cases of type 2 diabetes. The projected increase in PALY lost between 2020 and 2040 is mainly caused by a projected increase in the number of people with type 2 diabetes. Sensitivity analyses showed that lowering the incidence rate may substantially attenuate this increase, which calls for increased primary preventive efforts on the population level. Avoiding future PALY lost associated with diseases like type 2 diabetes is particularly warranted in light of a decreasing population proportion of working age [11]. From a fiscal perspective, required resources to achieve reductions in diabetes incidence and diabetes-related outcomes should be considered in light of the potential productivity revenue estimated in this study.

### Strengths and limitations

One strength of our study is that it combines a projection model for the future number of people with type 2 diabetes with measures of productivity, which allows us to project future productivity losses on the individual and population level. Furthermore, it allows us to estimate productivity in terms of PALY, which has the advantage of expressing various determinants of productivity (mortality, presenteeism, absenteeism, LFP) on the time scale [9, 10]. Using the time scale facilitates the intuitive interpretation of productivity losses and therefore the communication of our results to decision makers and people with type 2 diabetes. However, the projections required various assumptions and input data, some of which were backed up by strong evidence, whereas others were based on rather weak evidence. For the prevalence and incidence of diabetes and the *MRR* associated with diabetes, we drew on published estimates based on data including all people with statutory health insurance in Germany (approximately 90% of the population) [15–17]. These sources provided precise input values, with the drawback of only considering diagnosed diabetes documented in clinical practice and with no differentiation by diabetes type for the incidence rate and the *MRR*. Unfortunately, no German data on current trends in incidence and excess mortality were available. We assumed that the incidence rate remains constant, because evidence suggests heterogeneous trends in the incidence rate between countries [23]. In contrast, decreasing trends in excess mortality are consistently reported from various countries. Hence, we based trends in *MRR* on Danish data, assuming that geographical proximity and comparable healthcare systems result in similar trends. Input data for presenteeism, absenteeism and LFP were based on a meta-analysis. Although Bommer et al. [3] did not differentiate by diabetes type, the estimates may serve as acceptable approximations for type 2 diabetes, since studies investigating only type 1 diabetes were excluded from the meta-analysis. However, as with trends in the incidence rate and excess mortality, it would have been preferable to use representative German data instead, given country-specific labour markets. Lastly, potential differences in prevalence between the resident population and people migrating to Germany were not included in our model. However, the resulting bias is probably small because of the comparably low number of migrants, as has been shown in the context of dementia [14].

### Conclusion

Mean PALY lost per person of working age with type 2 diabetes in Germany in 2020 was 2.6 productive life years compared with a person of the same age and sex without type 2 diabetes. On the population level, productive life years lost amount to 12.1 million years in the population with type 2 diabetes. For 2040, PALY lost on the individual level are projected to remain similar to 2020, whereas PALY lost on the population level are projected to increase to 15.4 million years due to an increased number of people with type 2 diabetes. Increased efforts to reduce the incidence rate of type 2 diabetes and diabetes-related complications may attenuate the future productivity burden.

## Supplementary Information

ESM (PDF 909 kb)

## Data Availability

We only used data on an aggregated level. The data are publicly available from the sources referenced in the Methods section.

## Abbreviations

LFP: Labour force participation
*MRR*: Mortality rate ratio
PALY: Productivity-adjusted life years
PLW: Productivity loss weight
YLL: Years of life lost
YPL: Years of productivity lost
