# Supplementary material for: Productivity-adjusted life years lost due to type 2 diabetes in Germany in 2020 and 2040
Source: Diabetologia. 2021 Mar 4;64(6):1288–97. doi: 10.1007/s00125-021-05409-3 (PMC8099797; doi:10.1007/s00125-021-05409-3)
Supplement: Supplementary file 1 — (PDF 909 kb) [file 125_2021_5409_MOESM1_ESM.pdf]

Electronic Supplementary Material (ESM)

ESM Methods

Projection of type 2 diabetes prevalence

The model we used for the projection of prevalence is the illness-death model depicted in ESM Fig. 1. One model characteristic is that illness is binary-coded, which means that illness is either present (1) or absent (0). Furthermore, the model implicitly assumes that the prevalence of migrants equals the prevalence of the resident population. Although there is no evidence against this assumption, one could relax it by using the theory presented in Brinks & Landwehr [1]. However, it has been shown that migration in Germany happens on such a small scale that violations of this assumption will affect the results only negligibly [1]. There are no further implicit assumptions of the model. The inputs of the model are the incidence rate and mortality rates of people with and without diabetes. The output is the prevalence.

The illness-death model (figure S1) is governed by the following partial differential equation [1]:

$$\partial p = (1 - p) \times [i - p \times (m_1 - m_0)].$$

The parameter ∂p is the temporal change in prevalence, p is the prevalence, i is the incidence rate, m_0 is the mortality rate of people without diabetes and m_1 is the mortality rate of people with diabetes. All parameters in the equation depend on calendar time t and age a (ESM Fig.1). Due to data availability, $p \times (m_1 - m_0)$ was substituted with $\frac{p \times (MRR-1) \times m}{p \times (MRR-1)+1}$, which is mathematically equivalent. MRR is the mortality rate ratio defined as $\frac{m_1}{m_0}$. Solving the equation by integration with respect to t and a using input values for the right hand side yields the prevalence. In the current application, t covers the range from year 2015 to year 2089 and a covers the range from age 20 years to 69 years. The input data for the right hand side of the equation-are described in the main text and in figures S2-S5. Future values for the right hand side were based on the assumptions on future trends described in the main text.

The partial differential equation is equivalent to a Markov model with transition probabilities depending on age and calendar time [2]. Hence, it is a time-inhomogeneous Markov model. The Markov property of memorylessness is irrelevant for the model, because the partial differential equation also holds for semi-Markov models (i.e. for transition probabilities depending on disease duration) [3].

Calculation of productivity losses on the individual and population level

Labour force participation (LFP) as reported by Bommer et al. [4] refers to the population currently employed (full and part-time) as well as people currently unemployed, but actively seeking employment. The lower labour force participation among people with type 2 diabetes compared to without type 2 diabetes indicates that people with type 2 diabetes are more likely to drop out of labour force (e.g. due to disability).

ESM Results

ESM Table 1. Population-level productive life years lost due to excess mortality (years of life lost, YLL), reduced labor force participation, absenteeism and presenteeism (years of productivity lost, YPL) among people with type 2 diabetes in working age in 2040 assuming 0.5% annual decreases and increases in the incidence rate of type 2 diabetes. Productivity adjusted life years lost (PALY) are the sum of YLL and YPL.

	Decreasing incidence scenario			Increasing incidence scenario		
**Age group**	**YLL**	**YPL**	**PALY**	**YLL**	**YPL**	**PALY**
			**Women**			
20-29	0.05 (0.03-0.06)	0.83 (0.69-0.97)	0.87 (0.74-1.01)	0.05 (0.04-0.07)	0.98 (0.83-1.15)	1.04 (0.87-1.20)
30-39	0.09 (0.07-0.12)	1.30 (1.09-1.51)	1.39 (1.18-1.60)	0.10 (0.08-0.13)	1.51 (1.27-1.76)	1.61 (1.37-1.86)
40-49	0.15 (0.12-0.18)	1.84 (1.56-2.14)	1.99 (1.70-2.29)	0.17 (0.13-0.20)	2.12 (1.80-2.47)	2.29 (1.96-2.63)
50-59	0.17 (0.13-0.20)	2.26 (1.92-2.62)	2.43 (2.08-2.79)	0.19 (0.15-0.23)	2.62 (2.22-3.03)	2.81 (2.40-3.22)
60-69	0.06 (0.05-0.07)	1.38 (1.17-1.59)	1.43 (1.23-1.65)	0.06 (0.05-0.08)	1.58 (1.34-1.82)	1.64 (1.40-1.89)
**20-69**	**0.51 (0.40-0.63)**	**7.61 (6.45-8.84)**	**8.13 (6.93-9.34)**	**0.57 (0.46-0.70)**	**8.81 (7.46-10.24)**	**9.39 (8.00-10.80)**
			**Men**			
20-29	0.07 (0.05-0.09)	0.37 (0.30-0.43)	0.43 (0.36-0.50)	0.08 (0.06-0.10)	0.43 (0.35-0.51)	0.50 (0.42-0.59)
30-39	0.12 (0.10-0.16)	0.66 (0.55-0.78)	0.79 (0.67-0.91)	0.14 (0.11-0.18)	0.78 (0.65-0.91)	0.92 (0.79-1.06)
40-49	0.23 (0.18-0.28)	1.36 (1.14-1.58)	1.59 (1.36-1.81)	0.26 (0.20-0.32)	1.59 (1.34-1.85)	1.85 (1.59-2.11)
50-59	0.25 (0.19-0.31)	1.95 (1.65-2.25)	2.20 (1.89-2.50)	0.28 (0.22-0.35)	2.27 (1.92-2.61)	2.54 (2.19-2.89)
60-69	0.08 (0.06-0.09)	1.12 (0.95-1.29)	1.19 (1.02-1.36)	0.08 (0.06-0.11)	1.27 (1.08-1.46)	1.35 (1.16-1.55)
**20-69**	**0.74 (0.57-0.93)**	**5.46 (4.59-6.33)**	**5.46 (4.59-6.33)**	**0.84 (0.65-1.05)**	**6.34 (5.33-7.35)**	**7.17 (6.16-8.19)**
			**Overall**			
20-29	0.11 (0.08-0.15)	1.19 (0.99-1.40)	1.31 (1.10-1.52)	0.13 (0.10-0.17)	1.41 (1.17-1.66)	1.54 (1.30-1.79)
30-39	0.21 (0.17-0.27)	1.96 (1.64-2.29)	2.18 (1.86-2.51)	0.24 (0.19-0.30)	2.29 (1.91-2.67)	2.53 (2.15-2.91)
40-49	0.37 (0.29-0.46)	3.20 (2.70-3.73)	3.58 (3.07-4.09)	0.42 (0.33-0.52)	3.72 (3.14-4.32)	4.14 (3.55-4.74)
50-59	0.42 (0.33-0.51)	4.22 (3.58-4.88)	4.63 (3.98-5.28)	0.47 (0.37-0.58)	4.88 (4.15-5.65)	5.35 (4.60-6.11)
60-69	0.13 (0.10-0.16)	2.49 (2.12-2.88)	2.62 (2.24-3.01)	0.15 (0.12-0.18)	2.84 (2.41-3.29)	2.99 (2.56-3.43)
**20-69**	**1.25 (0.97-1.55)**	**13.06 (11.04-15.19)**	**14.31 (12.27-16.41)**	**1.41 (1.11-1.75)**	**15.14 (12.79-17.6)**	**16.55 (14.19-18.98)**

Numbers are in million years (95%-confidence intervals)

ESM Figures

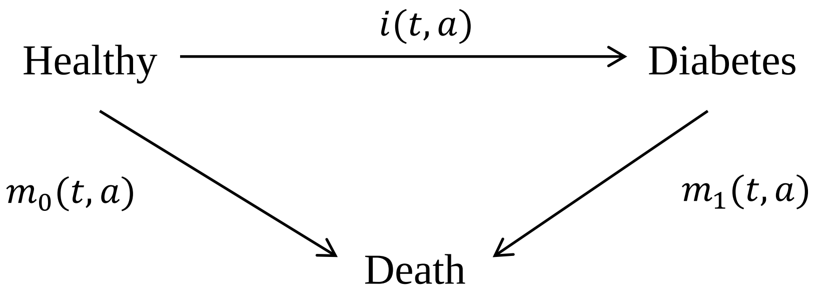

ESM Fig 1. Illness-death model

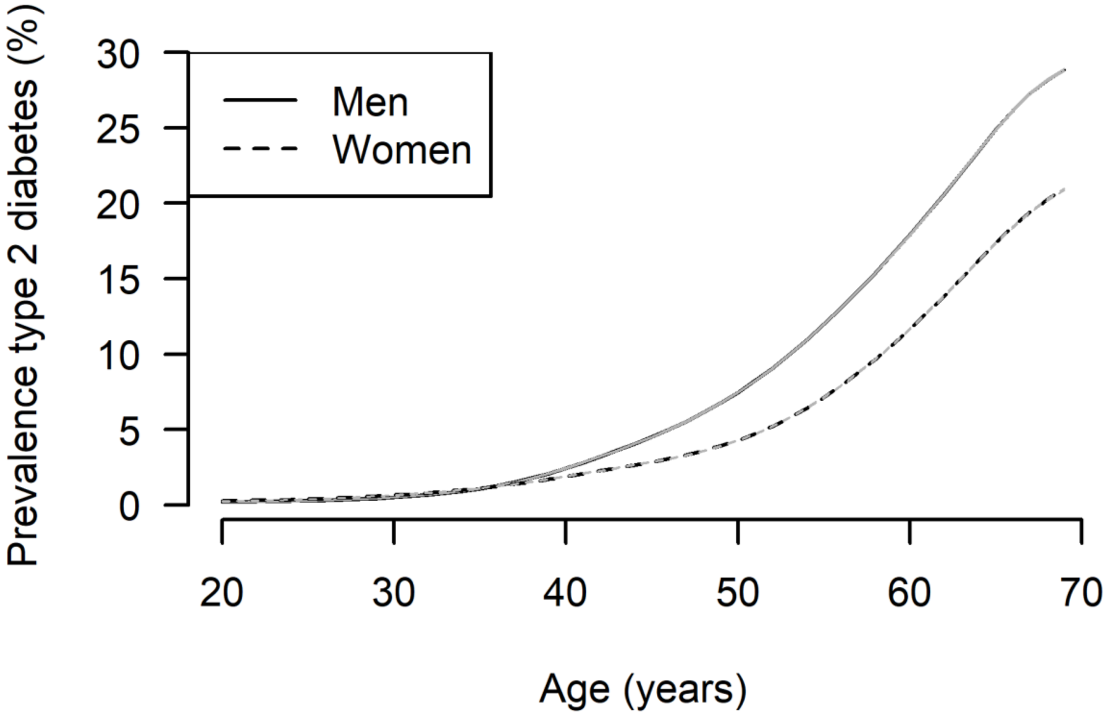

ESM Fig 2. Age-specific prevalence of type 2 diabetes in Germany in 2015. Grey lines represent 95%-confidence intervals. Own illustration based on [5].

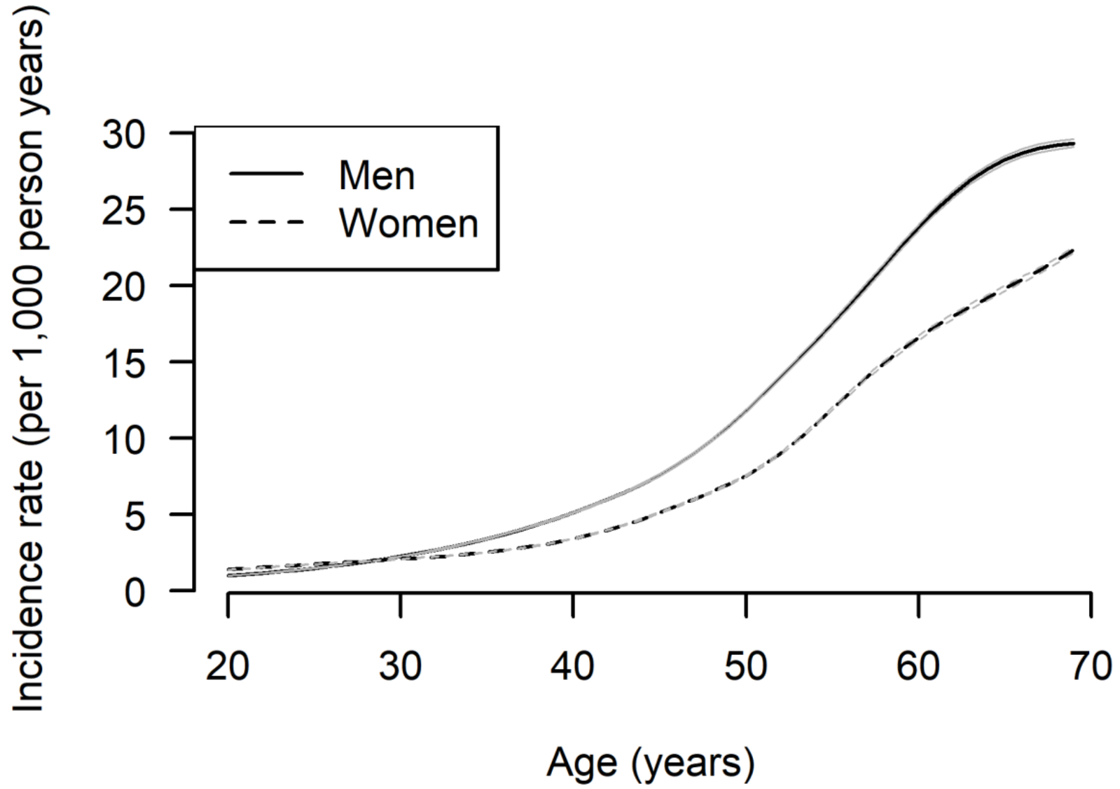

ESM Fig 3. Age-specific incidence rate of diabetes in Germany in 2012. Grey lines represent 95%-confidence intervals. Own illustration based on [6].

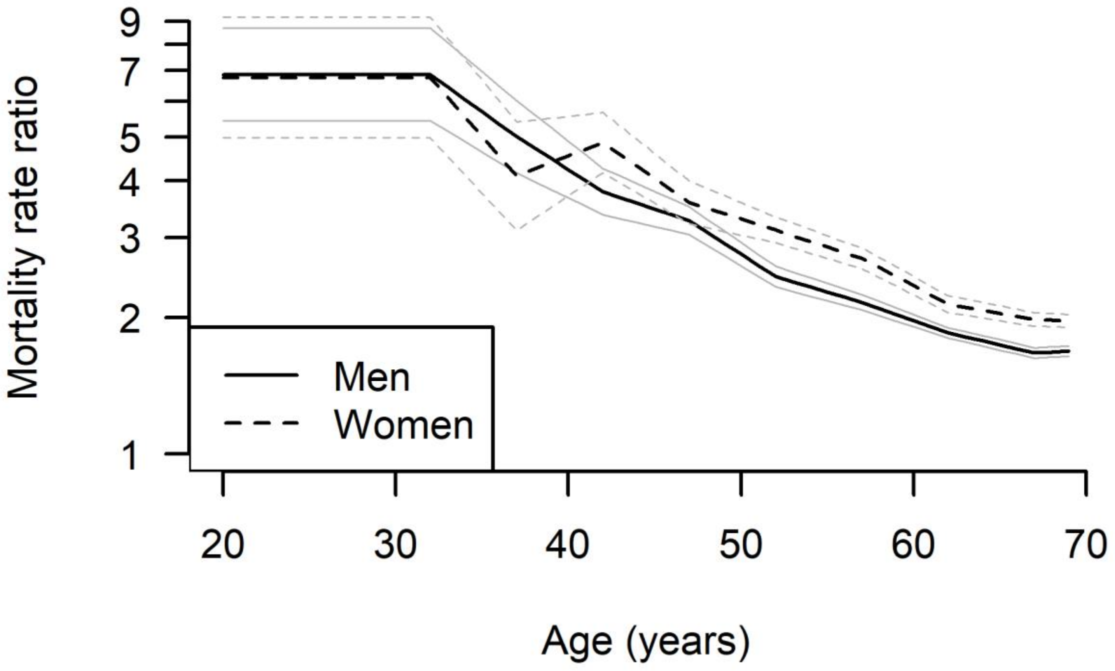

ESM Fig 4. Age-specific mortality rate ratio associated with diabetes in Germany in 2014. Grey lines represent 95%-confidence intervals. Own illustration based on [7].

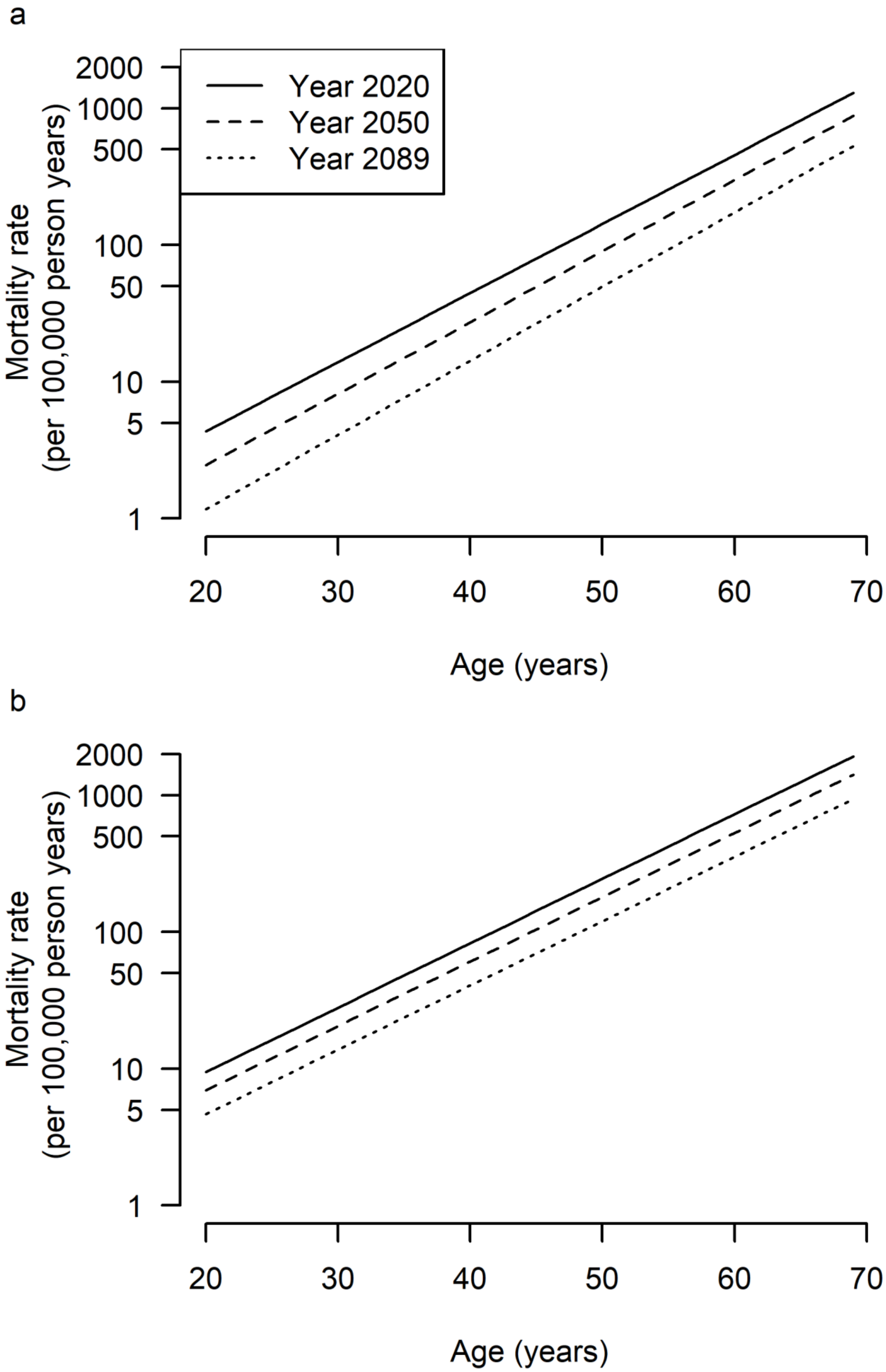

ESM Fig 5. Age-specific mortality rate of the population in Germany. Own illustration based on [8].

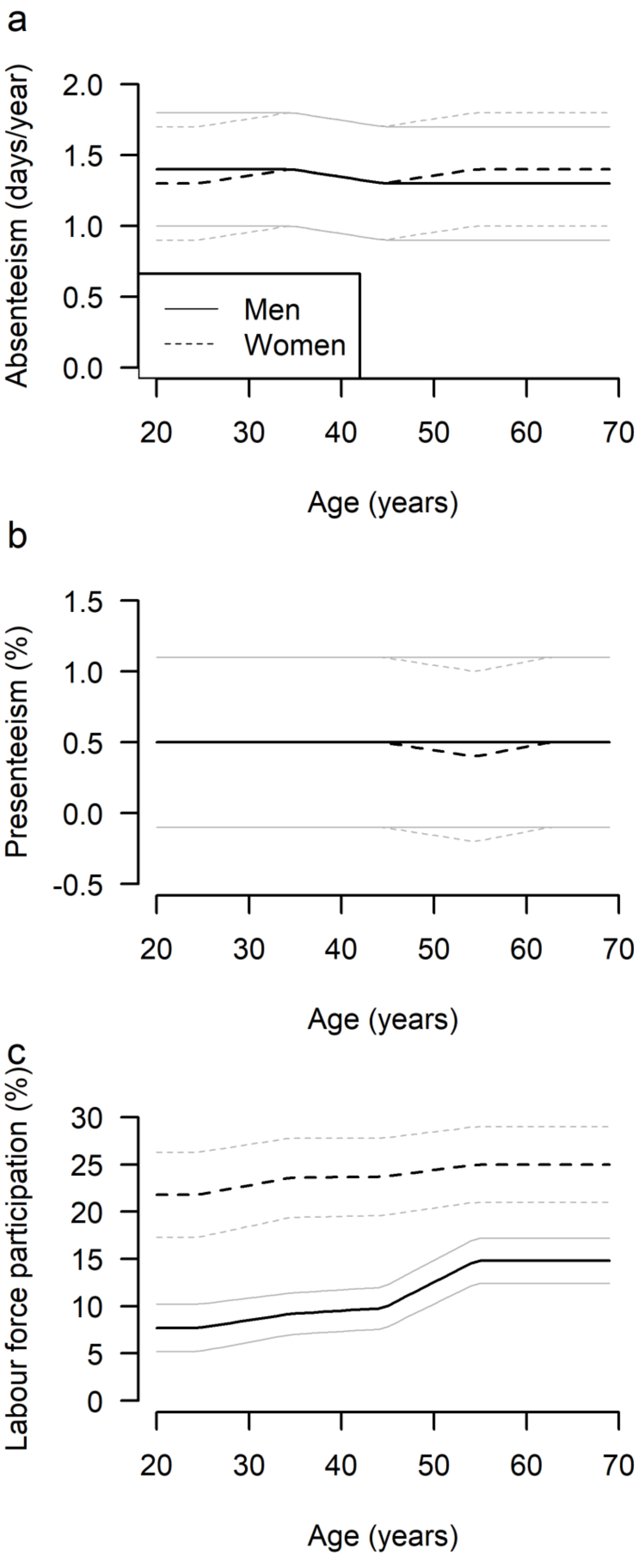

ESM Fig 6. Age-specific differences in productivity between people with diabetes vs. without diabetes with regard to absence days per year (a), productivity at work (b) and probability of participating in labour force (c). Grey lines represent 95%-confidence intervals. Own illustration based on [4].

References

[1] Brinks R, Landwehr S (2014) Age- and time-dependent model of the prevalence of non-communicable diseases and application to dementia in Germany. Theor Popul Biol 92: 62-68

[2] Brinks R, Hoyer A (2018) Illness-death model: statistical perspective and differential equations. Lifetime Data Anal 24: 743-754

[3] Brinks R, Landwehr S (2015) A new relation between prevalence and incidence of a chronic disease. Math Med Bio 32: 425-435

[4] Bommer C, Heesemann E, Sagalova V, et al. (2017) The global economic burden of diabetes in adults aged 20-79 years: a cost-of-illness study. Lancet Diabetes Endocrinol 5: 423-430

[5] Goffrier B, Schulz M, Bätzing-Feigenbaum J (2017) Administrative Prävalenzen und Inzidenzen des Diabetes mellitus von 2009 bis 2015. Versorgungsatlas-Bericht 17/03: 10.20364/VA-20317.20303

[6] Schmidt C, Reitzle L, Dreß J, Rommel A, Ziese T, Heidemann C (2020) [Prevalence and incidence of documented diabetes based on health claims data-reference analysis for diabetes surveillance in Germany]. Bundesgesundheitsblatt Gesundheitsforschung Gesundheitsschutz 63: 93-102

[7] National Diabetes-Surveillance at the Robert Koch Institute (2019) Diabetes in Germany – National Diabetes-Surveillance Report 2019. Available from http://diabsurv.rki.de, accessed 01 April 2020

[8] Federal Statistical Office (Destatis) (2019) 14th coordinated population projection. Available from https://www.destatis.de/DE/Themen/Gesellschaft-Umwelt/Bevoelkerung/Bevoelkerungsvorausberechnung/Publikationen/Downloads-Vorausberechnung/bevoelkerung-deutschland-2060-5124202199014.html, accessed 28 July 2020
